# Investigating How Bowel Cancer Survivors Discuss Exercise and Physical Activity Within Web-Based Discussion Forums: Qualitative Analysis

**DOI:** 10.2196/13929

**Published:** 2019-12-16

**Authors:** Alicia Olsen, Justin Keogh, Sally Sargeant

**Affiliations:** 1 Faculty of Health Sciences and Medicine Bond University Robina Australia; 2 Human Potential Centre Auckland University of Technology Auckland New Zealand; 3 Cluster for Health Improvement Faculty of Science, Health, Education and Engineering University of the Sunshine Coast Sunshine Coast Australia; 4 Kasturba Medical College Mangalore Manipal Academy of Higher Education Manipal India; 5 School of Health and Human Sciences Southern Cross University Bilinga Australia

**Keywords:** exercise, physical activity, cancer, qualitative research, patient portals, internet

## Abstract

**Background:**

Online cancer support group discussions enable patients to share their illness experience with others. The sharing of technical and emotional support information and the ability to ask for advice are some of the primary discussions shared online. People with bowel cancer can also use these forums to support each other by sharing information based on personal experiences. This type of support provides newly diagnosed patients with advice about several topics, including exercise from those who have been there. Information gathered from online discussion boards may complement the advice received by health professionals.

**Objective:**

This study aimed to explore the nature of information related to exercise and physical activity exchanged online for cancer survivors.

**Methods:**

A public open access bowel cancer discussion board was searched for threads containing information related to physical activity or exercise. Keywords such as *exercise*, *physical activity*, *moving*, *walking*, *lifting*, *weights training*, and *resistance* were used to search for threads (online conversations) related to exercise or physical activity. Only threads initiated by bowel cancer patients or survivors were included. From more than 6000 posts, the inclusion criteria yielded 75 threads for analysis. Inductive thematic analysis was conducted across all included threads.

**Results:**

Analysis yielded 3 main themes: *level of exercise competence*, *beneficial dimensions of exercise*, and *faith in the knowledge*. *Level of exercise competence* illustrated the varying definitions of exercise that members of the forum discussed in the forum. *Beneficial dimensions of exercise* revealed that forum members shared both the spiritual benefits associated with exercise as well as the physical benefits or goodness that they feel exercise or physical activity provides them. *Faith in the knowledge* of exercise demonstrated that forum members were aware of the general benefits of exercise but felt disappointed that it did not keep the cancer at bay. However, members also had faith that exercise would keep them healthy after diagnosis and treatment.

**Conclusions:**

The analysis revealed that people with bowel cancer discuss exercise and physical activity online and that they view exercise as having a mostly positive influence on their cancer journey. However, personal definitions of exercise became a source of conflict within the group. People with bowel cancer seeking information about exercise may benefit from participating in online support groups as it appears that there are many similar others willing to share their personal experiences with exercise. In addition, health care professionals responsible for caring for people with bowel cancer may use these findings to discuss exercise with their patients while being mindful of how they may view exercise.

## Introduction

### Background

Many patients with chronic disease increasingly utilize online health support groups, with the Pew Research Center reporting that 20% (N=2253) of internet users living with a chronic disease participated in some form of online discussion forum [1]. Specifically, people diagnosed with cancer use the internet to search for disease-related information, for supportive communication, for practical tips on daily living with cancer [2-4], to find out more about their disease as well as other general health issues [5], and to search for information regarding diagnosis and treatments [6].

The value of advice received from online cancer support group members appears to be different from the value of advice received from health care professionals. In some cases, the information sought in online cancer support discussion boards may complement the advice received from the health professionals [7]. However, the influences of other cancer patients’ experiences discussed in online forums were found to be greater than the influences of advice given by doctors [8]. Such a view is consistent with the findings of Gill and Whisnat [9] who reported that people with ovarian cancer commonly used online forums to discuss whether the health information received from their health care professionals was honest and accurate. The members used online forums when their trust in the health care professional was low, such as in the case where the participant was not satisfied with the health care professional’s suggestion to wait before initiating treatment. They also shared information about diet, activities related to daily life, and treatment side effects [9]. It is, therefore, suggested that online cancer forums provide a platform for people to discuss and advise each other on issues related specifically to their cancer, especially when they do not feel that they are getting the required information and support from their health care professionals.

Analyses of online discussion forums reported that forums served as an opportunity for members to communicate their experiences with similar others when they may have not been able to speak to others directly [10-12]. This included the ability to discuss intimate bodily symptoms [13]. Men with prostate cancer reported being able to discuss their uncomfortable and challenging experiences, such as urinary and bowel function and continence, with other members of the group [14].

Courneya and Friedenreich [15] noted that 40 out of 130 (31%) cancer survivors maintained and 21 out of 130 (16%) returned to physical activity following treatment completion. Furthermore, it was suggested that many have searched the internet and online groups for information about exercise specific to their form of cancer and treatment [16]. This implies that people with cancer are seeking exercise information, support, and advice from online cancer support groups. However, relatively little research has examined how individuals with bowel cancer may interact and benefit from online communities, despite bowel cancer being the third most diagnosed cancer internationally [17]. Despite the frequency of its diagnosis and relatively high survival rates, bowel cancer commands considerably less public—and scholarly—attention in contrast to the other 2 leading cancers, breast and prostate cancers [18].

### Objectives

Research advocates physical activity and exercise for people with bowel cancer [19-21], yet little is known about bowel cancer patients’ experience with physical activity, which makes it challenging for health care professionals to provide effective physical activity counseling to this population. This study applies a qualitative analysis of messages exchanged online to explore how bowel cancer online forum members define exercise in addition to describing their previous experiences with exercise. The aim of this study was to provide insights from the bowel cancer patients’ perspective that can allow health care professionals to better counsel and support their patients with respect to their exercise and physical activity needs.

## Methods

### Selection of Online Discussion Group

To illustrate the views of people with bowel cancer about exercise, a qualitative inductive thematic analysis (ITA) of online asynchronous archived discussions on a bowel cancer discussion board was conducted. A complete search of online bowel cancer discussion groups was conducted using the Google search engine using the keywords *bowel cancer*, *online forum,* and *online support group*. The forum chosen for analysis is one of the largest online cancer forums [22] and is available internationally. International availability meant a greater potential of reaching a wider demographic. Another reason for choosing this forum was that it clearly specifies that any information posted on the discussion boards is open to public view.

An asynchronous discussion board was chosen because this type of communication enables individuals to connect with other members at a time and place convenient to them [23]. In addition, posting on an asynchronous discussion board allows all members to potentially read and respond to a message thread, even though it may have been originally posted many days or weeks previously. Furthermore, participants may search message threads using keywords to locate and read information relevant to their current information needs, with these individuals being the primary users of discussion boards [24].

The discussion boards are open to people currently being treated for bowel cancer; survivors of bowel cancer; and caregivers, significant others, and other family members of people with cancer. The research question sought to answer how people with bowel cancer discuss exercise; therefore, threads initiated by patients and survivors of bowel cancer only were included in the analyses. Threads that did not explicitly include information about the initial post’s connection to bowel cancer were not included in the analysis. Inclusion criteria required that forum posts (1) must be initiated explicitly by a person currently or formally diagnosed as having bowel cancer and (2) must include a discussion related to physical activity or exercise.

### Ethical Considerations

This research was approved by the lead university’s ethics committee.

Traditional research ethics dictate that researchers must take the necessary precautions to ensure the privacy and confidentiality of data as well as collection of informed consent of all participants [25]. It is argued that information posted in a public discussion group is a public act and, therefore, is available for public consumption, which requires no more than the usual precautions [26]. Eysenbach and Wyatt [27] report that a *passive analysis* of online data collection does not mandate informed consent. A *passive analysis* occurs when the researcher strictly observes the interaction and communication of a public online space. This research did not actively engage with members of the online community, meaning that the processes involved in the study could be classified as a *passive analysis* of the data.

### Data Collection

At the time of data collection, the discussion board consisted of 280,356 posts. Keywords such as *exercise*, *physical activity*, *moving*, *walking*, *lifting*, *weights* *training*, and *resistance* were used to search for threads related to exercise or physical activity. Threads identified were then opened, and the posts were read. *Search by Keyword* identified keyword search terms within posts. *Search by Title* identified keyword search terms in the title of the threads.

Each thread contained its own unique title named by the member who initiated the thread. This title was used as an indicator by other members to identify the contents of the thread. Each thread was briefly read online to establish that it met the inclusion criteria. Once all threads had been reviewed, they were organized chronologically based on the date of the initial post of the thread. The lead researcher (AO) read through all threads and posts several times to familiarize herself with the general context of the posts, the online community, and the interactions within the online community. Threads were dated from March 4, 2003, with the earliest thread related to physical activity or exercise, to July 11, 2014, when data collection ceased. Date restrictions on threads were not warranted because knowledge of the benefits was available as of 1999 when Courneya [28] published one of the first studies investigating the benefits of exercise for people diagnosed with cancers other than breast cancer.

Discussion threads initiated by significant others, caregivers, or others were excluded. A thorough search of the online discussion board yielded 139 threads, with 37 threads identified by a title search and an additional 102 threads by a keyword search.

### Data Analysis

An ITA was conducted following the recommendations of Braun and Clarke [29]. ITA is a systematic approach that identifies commonalities laterally across the dataset and assists with outlining an understanding of what those commonalities indicate.

### Analytic Approach

Highlighting phrases and statements within the text referring to exercise and physical activity achieved the first step of analysis. Initial notes were then made from the phrases and statements. Initial notes from the data extracts were then grouped based on similarities into initial codes. The initial codes were then analyzed and grouped together to create provisional themes. Provisional themes were created from similar initial codes, opposing initial codes, and linearly across a theme to create levels of a particular theme.

Regular meetings were held between the lead author and coder and an experienced qualitative researcher (SS), where potential provisional themes were discussed based on the initial codes to ensure a thorough analysis of the extracts. The lead coder also completed self-reflections to identify possible biases and address personal thoughts and actions during the analytical process. Following the recommendations of Braun and Clarke [29], the provisional themes were then checked to ensure that they were representative of the dataset and the general narrative of the analysis. Initial notes and initial codes were developed across 96 threads with 331 unique contributors, where data saturation was deemed to have occurred as reading further posts did not yield any new significant themes [30].

## Results

A total of 3 themes, *level of exercise competence*, *beneficial dimensions of exercise*, and *faith in the knowledge of the benefits of exercise* were identified within the online forum that illustrated the role and presence of exercise for people with bowel cancer.

### Theme 1: Level of Exercise Competence

Level of exercise competence indicated that members of the forum held different ideas of what defined exercise, what exercise provided a health benefit, and what was an appropriate amount of exercise. One post in the forum said:

People can have work that requires difficult physical labor for decades and still have their health deteriorate as they age, because they haven’t been doing the right kinds of exercise. I watched my mother’s health go in two years, despite her active life and very healthy eating habits—she gardened and did yard work, and walked, but she never ran, or cycled, or swam.

Participant 80

This member believed that there were the *right kinds* of exercise such as running, cycling, or swimming that preserved health, when in fact gardening, yard work, and walking can improve health outcomes [31,32]. Some members had a jaded view on what type of physical activity could help recovery and held the belief that because it was not the *right kinds* of exercise, there would be no benefits. This belief encouraged feelings of helplessness of other members because it pushed the view that running, cycling, or swimming were the only beneficial forms of exercise and physical activity for people with bowel cancer.

The description of what does, or does not, constitute as exercise was commonly discussed among members of the group. One forum member wrote:

I am trying to find “the perfect” diet and exercise plan so I can help my body beat this and recover.

Participant 141

This suggested that this participant was feeling stressed with the idea of constructing *the perfect* survivorship plan, a feeling intensified by posts that discussed the right kinds of exercise. This pressure may have discouraged this participant from beginning any activity because the plan might have been viewed by others as suboptimal or even flawed because it did not meet the other’s standards of what constituted appropriate exercise.

This view of what constituted appropriate exercise was not held consistently across the group. For example, one member wrote:

I try to swim and do exercises 5 times a week, can’t run or anything else but it all helps.

Participant 269

This member indicated that they participated in some form of structured exercise but also believed that any little bit counted even if participation in other forms of structured exercise was limited. Another member wrote:

We try to have Friday night date night at a local restaurant and we walked there. Hey, it’s something!

Participant 105

This member accepted that although there was no engagement in any vigorous form of exercise or physical activity, something was achieved, which was felt to be better than nothing.

### Theme 2: Beneficial Dimensions of Exercise

This theme illustrated that bowel cancer patients discussed exercise as having many benefits. One member wrote:

Another thing that is SO emphasized by every doctor I’ve seen or any medical article I’ve read is exercise. I’m not as good about it as I should be, but when I do walk on a regular basis, I feel better, both physically and mentally.

Participant 105

This member demonstrates to other members of the forum how exercise, specifically regular walking, has provided them with benefits. To corroborate the benefits of exercise they experience, they have included hearing about the benefits of exercise from additional sources, namely, doctors and medical articles. Another member wrote:

I too am and was a fitness freak (running/walking/weights/yoga daily) and almost went insane after my surgery not being able to do as much...but, I am now back to almost my normal routine and it makes a HUGE difference in how I am feeling both mentally and physically.

Participant 13

This member illustrated to the group how being fit and engaging in various fitness activities has provided them with general mental and physical benefits. Furthermore, this member highlights the magnitude of these benefits.

One subtheme, called spiritual benefits of exercise, was expressed by using phrases that illustrated a heightened sense of unworldliness. In their own way, bowel cancer survivors were embracing the spirituality and peace that exercise offered them and shared these experiences with other members of the group. For example, one member stated:

The walking will calm the universe.

Participant 255

Members of the forum also discussed that physical activity has the ability to provide inner strength and tranquility. For example, 1 contributor wrote:

I always moved as much as I could, and the pleasant fatigue and peace that I gained from the daily exercise helped me sleep and feel good about my day.

Participant 76

This member illustrates how exercise provided serenity that translated into changing their daily mood. This commentary highlights the psychological importance of exercise and physical activity.

Another subtheme, physical benefits of exercise, indicated the benefits exercise had on technical aspects of cancer and was expressed by this member who said:

We also exercise most every day and participate in yoga several times a week. I really believe that all this has helped me manage side effects, reduce tiredness, rebound to feeling “normal” again, etc.

Participant 208

This member shared with others that exercise was helping with cancer treatments. The owner of the post expressed the value exercise had for them during their treatment because it was helping to decrease the feelings of fatigue and allowing feelings of normalcy. Here, this member was having a better response to the disease and the treatments because of exercise. Other members shared the exercise benefits they received in a more general sense:

I was encouraged to walk a lot after the surgery, and I did—I think this helped my recovery.

Participant 22

This post demonstrated that forum members had positive experiences with exercise in relation to bowel cancer and were sharing these experiences with other members of the group.

The final subtheme, medical benefits, illustrated that some members of the forum talked about exercise as a form of complementary medicine or the medical benefits and acknowledged that:

While it may feel that your wife is ratting on you, I think that she is aware that walking is the best medicine for you. I imagine your surgeon told you that too.

Participant 144

Here, this member related exercise to a form of medicine or treatment for bowel cancer*.* A different member shared the same view and wrote:

I view my exercise and my diet and meditation as an extension of my treatment.

Participant 255

Members posted about exercise in medical terms by using words such as *medicine*, *treatment*, *holistic*, and *vital.* The members were using these terms to describe the value exercise had during and following treatment. The use of the medical terms suggested that these members value exercise and physical activity as therapeutically important to their recovery and had adopted a more active lifestyle as part of their medical treatment in the same way as one undergoes chemotherapy or radiotherapy as a form of therapy.

One member of the forum illustrated how they viewed exercise as a part of their medical journey. This member stated:

It may be hard to believe when you’re lying in pain but the walking and other exercise are so good (not to mention vital) to your recovery.

Participant 144

This member states the indispensable importance of exercise in recovery and survivorship. This member explains that being physically active is the best thing one can do. From the analysis of the threads, it is uncertain whether or not members of the forum discussed exercise in medical terms because of the influence of the interaction they had with their health care professional. However, as some members of the forum were encouraged to participate in physical activities by their health care professional, it is possible that when they shared this information with the forum, they adopted terminology used by their health care professional. This may have resulted in other members relaying that advice to others.

### Theme 3: Faith in the Knowledge of the Benefits of Exercise

This theme describes some members’ disappointment in their belief that exercise would have kept them cancer free, but at the same time, speaks about the faith they have in the ability of exercise to help the recovery process. A member felt particularly let down and voiced skepticism about published research based on their experiences. This member writes:

Thank you for an interesting item, I have to admit I look at these studies with a jaded eye now. I was exercising (brisk walking or swimming, strength training and yoga) regularly for years (20 or more) and eating my nine servings of veggies/fruit per day. My co-workers would make fun of me. I ate very little red meat (Once a month or less). Yet I still came done with stage 4 rectal CA.

Participant 48

This member expressed feelings of defeat, which may have had negative implications for whether this person would continue to have an active lifestyle after treatment and may have influenced any future discussions about activities with either other members or health care professionals. This member had negative feelings toward exercise. Some other members of the forum expressed similar sentiments. Other members wrote:

I too was very active, walking 5 miles each morning, eating healthy—all the things I thought would keep me healthy—luck of the draw I guess.

Participant 7

A different forum member wrote:

It’s funny though, you take care of yourself, try and eat right most of your life, work out physically and cardio, maintain your weight throughout your life and blamn, 2006 cancer and 2010 could be looking at a pace maker in my retirement years. Then you have people that are overweight, obese, don’t watch what they eat, don’t exercise, smoke, drink etc., and blamn, never a health issue. Scratch head and go figure :) :) :) :) LOL!!!!!!!!!

Participant 83

However, other members of the forum did not appear to adopt these negative feelings about why being physically active had not prevented them from being diagnosed with bowel cancer. Some members of the forum shared that they too exercised before diagnosis, but they did not blame exercise for not keeping them cancer free. One member wrote:

I have exercised most of my life before (not much during) and after cancer. Even if it didn’t help stop the beast, which I do believe it does, I would still do it.

Participant 103

This member chose to have a positive outlook and had accepted that sometimes bad things happen, but this did not change their attitude about exercise. Another member wrote:

My doctor told me there would be a 95% chance that the cancer would return. I asked him what I could do to prevent reoccurrence. He told me exercise. That was no problem for me as I have always exercised.

Participant 239

This member appeared to have taken things as a matter of fact. It would seem that diagnosis had been accepted, treatment had been completed and now this member was working on surviving. According to their doctor, exercise was the way to survive. This member may not know that exercise had been reported to reduce the risk of bowel cancer and, therefore, did not express feelings of defeat, or this member did know, but had chosen to move forward in survivorship without dwelling on what had already happened regarding the cancer diagnosis.

## Discussion

### Principal Findings

An ITA of an online asynchronous bowel cancer discussion board identified several ways in which exercise and physical activity are present in the lives of people diagnosed with bowel cancer. Furthermore, this analysis identified ways in which they viewed exercise and physical activity and how they communicated their views on this topic with each other.

### Limitations

Some limitations of this study exist, with the first being that the keywords related to other forms of exercise including tai chi, yoga, or Pilates were not explicitly searched. However, the search terms that were used did identify threads in which physical activities including yoga, tai chi, and Pilates were discussed among the forum members. Furthermore, the evidence surrounding the benefits of activities such as yoga for people with bowel cancer is mostly inconclusive. A randomized control trial reported that yoga did not have a significant effect on the quality of life of people with bowel cancer [33]. The second limitation is the potential cultural difference between those on the online forum. It has been reported that Puerto Rican breast cancer survivors preferred to not use the internet for seeking information on physical activity [16]. Similarly, African Americans and Latinos aged 50 years and above were more likely to search the internet for bowel cancer information compared with whites aged 50 years and above who utilized the internet to search for general health information [34]. Third, not all bowel cancer discussion forums will serve the same purpose. An evaluation of a different online bowel support community reported that threads were initiated by asking questions as a way to initiate conversations [11]. Although this was similar to the online support community analyzed here, there were several posts wherein members would introduce themselves to the group along with information about who they were, what their diagnosis was, and any therapies they were currently using or thinking about using. Therefore, the themes discussed in this research may only be applicable to this online support community. Although a limitation, these themes are apparent in the threads and speak about the ways some bowel cancer patients discussed exercise and physical activity among themselves in the online medium. They can, therefore, still inform health care professionals about the way exercise is discussed among some bowel cancer patients and can help inform future physical activity counseling practices and survivorship guidelines.

Turner et al [10] reported that members who spent more time reading posts on online communities had limited traditional face-to-face partner support. It could be speculated that more time spent reading would coincide with more posts. However, this was not the case, with Turner et al [10] finding no significant interaction between depth of face-to-face support or depth of online support and posting frequency. The nature of this analysis was to review discussions of exercise and/or physical activity; therefore, particularly passionate members of the group may have increased the likelihood of introducing user frequency bias simply by being more communicative than other members about the topic. This, however, speaks about the passion some bowel cancer survivors have in relation to exercise and physical activity and the important role exercise played in their survivorship plan.

### Comparison With Previous Work

This analysis indicated that exercise and physical activity had many different meanings to members of this forum. This was identified by the *level of exercise competence* theme. While discussing exercise, which had no clear and uniform meaning across all forum members but quite narrow definitions for some individuals, the lack of nonverbal cues and tone of voice within the online environment meant that there were numerous instances of negative emotions and animosity between members of the forum. The scope of what exercise is and is not to individuals with bowel cancer was an important finding of this research. First, because it clarified for researchers that more research remains to be done to further educate bowel cancer patients on what constitutes exercise and physical activity. Furthermore, the difference in understanding the definition appears to influence their exercise and physical activity habits, which may have implications on how health care professionals gather information from and educate their patients during discussions related to cancer survivorship. As lack of time was indicated as the primary barrier to discussing exercise with cancer patients [35], developing an efficient way to gain a mutual understanding of the definition of exercise with each patient may be important to the efficiency of care.

The varied opinions of the members on what activities were considered exercise had implications for whether or not other members engaged in physical activity or contemplated engaging. Understanding that all forms of physical activity no matter how brief or low intensity are likely to have some benefits for insufficiently active cancer survivors and that some activity is better than none would encourage the discouraged to participate at some level. This has the potential to lead to greater health benefits from future involvement in longer-duration and/or higher-intensity physical activities. However, it should also be noted that there is the potential for harm in sourcing exercise advice and information from peers or those not trained in prescribing exercise. For example, it is possible that underactive or deconditioned members of the online forum may be at risk of a musculoskeletal or cardiovascular event if they begin exercise programs beyond their capabilities. At present, the negative outcomes of peer advice on exercise have not been explored; therefore, it is not possible to draw any definitive conclusions on the relative potential of benefit versus harm from seeking peer or online advice.

Exercise provided different benefits to those who chose to participate in exercise, and this information was shared among the members of the online forum. The *beneficial dimensions of exercise* theme identified that members of the forum categorized these benefits as spiritual, physical, and medical benefits. Similarly, women diagnosed with gynecological cancer used exercise as part of their spiritual practice [36]. However, because of the quantitative nature of Lopez et al’s study [36], the significance of exercise in relation to life meaning was not explored. From the analysis of the online forum data in this study, it can be speculated that exercise provided these bowel cancer patients with a feeling of purpose. Members of this forum discussed the ability of exercise to reconnect them with life and feelings of calmness experienced following exercise. People diagnosed with bowel cancer who participated in preoperative exercise reported that exercise gave them a sense of purpose and that this assisted with their ability to perform their usual activities [37]. Members of this online forum wrote about similar relationships in their posts to each other about the spiritual benefits of exercise.

The members of the forum also discussed the physical benefits of exercise. Similar findings have been reported elsewhere. For example, a qualitative inquiry into the exercise experiences and preferences of bowel cancer survivors, who completed a 12-week individualized exercise intervention, reported significant improvements in strength, aerobic fitness, and endurance [38]. The reported physical benefits of the 12-week exercise intervention were similar to the benefits members discussed in this online discussion forum. This is an expected finding based on the documented results, which suggested that exercise and physical activity have many positive physical benefits for people diagnosed with bowel cancer. The findings of this study, therefore, add to the existing evidence with respect to how the benefits obtained by people with bowel cancer may reflect how they define and view exercise as a part of their cancer survivorship.

One unique finding of this analysis was the language members of the forum used when discussing exercise in terms of medical benefits. The forum members used terms generally reserved for standard cancer care. It was referred to as an *extension of treatment* or *different side of treatment* and was understood by this online bowel cancer community as not universally accepted by the medical community as standard or usual care. This information adds to the understanding of the role of exercise in cancer care and survivorship from the patients’ perspectives. It is possible that health care professionals hold similar views based on discussions the bowel cancer community had about the role of their health care professional in discussing exercise with them. However, more exploration is needed. The benefits of exercise reported by the patients in the online discussion forum were influenced to some extent by the patients’ varied definitions of exercise, but unified by the variety of benefits experienced by physically active members of the forum.

This analysis recognized that exercise was an important component in the lives of many people diagnosed with bowel cancer. This was expressed within the third theme, f*aith in the knowledge of the benefits of exercise.* Forum members shared their negative and positive experiences and feelings toward exercise with each other in the online discussion board. This theme explained that people diagnosed with bowel cancer have a complicated relationship with exercise, with several members expressing their anger toward the inability of physical activity and exercise to keep them disease free, whereas others shared their belief that exercise would help them in their recovery. Shaha and Cox [39] reported that people receiving a bowel cancer diagnosis experienced fear and anxiety, which led them to question their choices, goals, and attitudes toward life. This level of anxiety was similar to what was demonstrated in the third theme in this study because members of the forum questioned their attitudes and beliefs about the goal they had set for themselves to live a healthy life through exercise and physical activity. To them, bowel cancer challenged this goal by way of threatening their identity. However, these feelings were not uniform for all members. This other group of bowel cancer survivors chose not to question their beliefs about exercise and continue exercising through the illness experience. In this way, the role of exercise transformed from reducing disease risk to improving the illness experience. These members trusted exercise to help them move forward. Such divergence of patients’ perceptions around the role of exercise and physical activity in reducing the risk of developing bowel cancer and improving their life post cancer diagnosis further supports the importance of health care professionals understanding their patients’ perceptions on exercise if they wish their patients to optimize their cancer survivorship.

To summarize, this study adds to the body of knowledge surrounding the ways in which bowel cancer patients discuss exercise and physical activity with their peers using a qualitative analysis of their discussions in an online forum. This study draws attention to the value bowel cancer patients place on exercise in their cancer journey as well as the way they are sharing that information with others. This study is novel in the examination of language in the online context in connection with bowel cancer patients’ discussions of exercise and physical activity. The results of this study can be used to inform health care professionals about how their patients view exercise and physical activity and where they are sourcing information related to this topic. Furthermore, such information can augment behavior change interventions relative to exercise and bowel cancer.

### Conclusions

In conclusion, the analysis of the public online asynchronous discussion board revealed that many members associated exercise with positive feelings and were aware of and enjoying the benefits of exercise in their recovery and survivorship. It was also apparent that other members were perturbed by the discussions and the meaning of exercise for them, with such discussions being perceived as negative and threatening.

The results also highlight some key features about the ways bowel cancer survivors and patients discuss exercise among themselves and the perceived role exercise has in recovery. For example, exercise played a large role in the recovery of many of the forum members. This was demonstrated in their posts about the many benefits they received from being physically active during their journey in addition to the faith they expressed in exercise helping them live better.

The themes identified within this analysis provide important insights into how people with bowel cancer experience exercise in relation to disease and the important role of exercise in survivorship. This information is important to clinical discussions about the role that exercise may play in bowel cancer survivorship and how the patients’ perceptions and previously held beliefs may influence their future exercise and physical activity behavior. Such insight may also influence future exercise and physical activity intervention research in terms of communication styles and techniques of physical activity counseling.

## Abbreviations

ITA: inductive thematic analysis
